# Predicting chronological age of 14 or 18 in adolescents: integrating dental assessments with machine learning

**DOI:** 10.1186/s12887-024-04722-1

**Published:** 2024-04-10

**Authors:** Shihui Shen, Yibo Guo, Jiaxuan Han, Meizhi Sui, Zhuojun Zhou, Jiang Tao

**Affiliations:** 1 Department of General Dentistry, Shanghai Ninth People’s Hospital, Shanghai Jiao Tong University School of Medicine; College of Stomatology, Shanghai Jiao Tong University; National Center for Stomatology; National Clinical Research Center for Oral Diseases, Shanghai Key Laboratory of Stomatology; Shanghai Research Institute of Stomatology, Shanghai, China; 2 Department of Stomatology, Kashgar Prefecture Second People’s Hospital, Kashgar Xinjiang, China

**Keywords:** Personal identification, Age determination, Machine learning, Dental age, Periodontal ligament visibility

## Abstract

**Aim:**

Age estimation plays a critical role in personal identification, especially when determining compliance with the age of consent for adolescents. The age of consent refers to the minimum age at which an individual is legally considered capable of providing informed consent for sexual activities. The purpose of this study is to determine whether adolescents meet the age of 14 or 18 by using dental development combined with machine learning.

**Methods:**

This study combines dental assessment and machine learning techniques to predict whether adolescents have reached the consent age of 14 or 18. Factors such as the staging of the third molar, the third molar index, and the visibility of the periodontal ligament of the second molar are evaluated.

**Results:**

Differences in performance metrics indicate that the posterior probabilities achieved by machine learning exceed 93% for the age of 14 and slightly lower for the age of 18.

**Conclusion:**

This study provides valuable insights for forensic identification for adolescents in personal identification, emphasizing the potential to improve the accuracy of age determination within this population by combining traditional methods with machine learning. It underscores the importance of protecting and respecting the dignity of all individuals involved.

**Supplementary Information:**

The online version contains supplementary material available at 10.1186/s12887-024-04722-1.

## Introduction

**Fig. 1:**
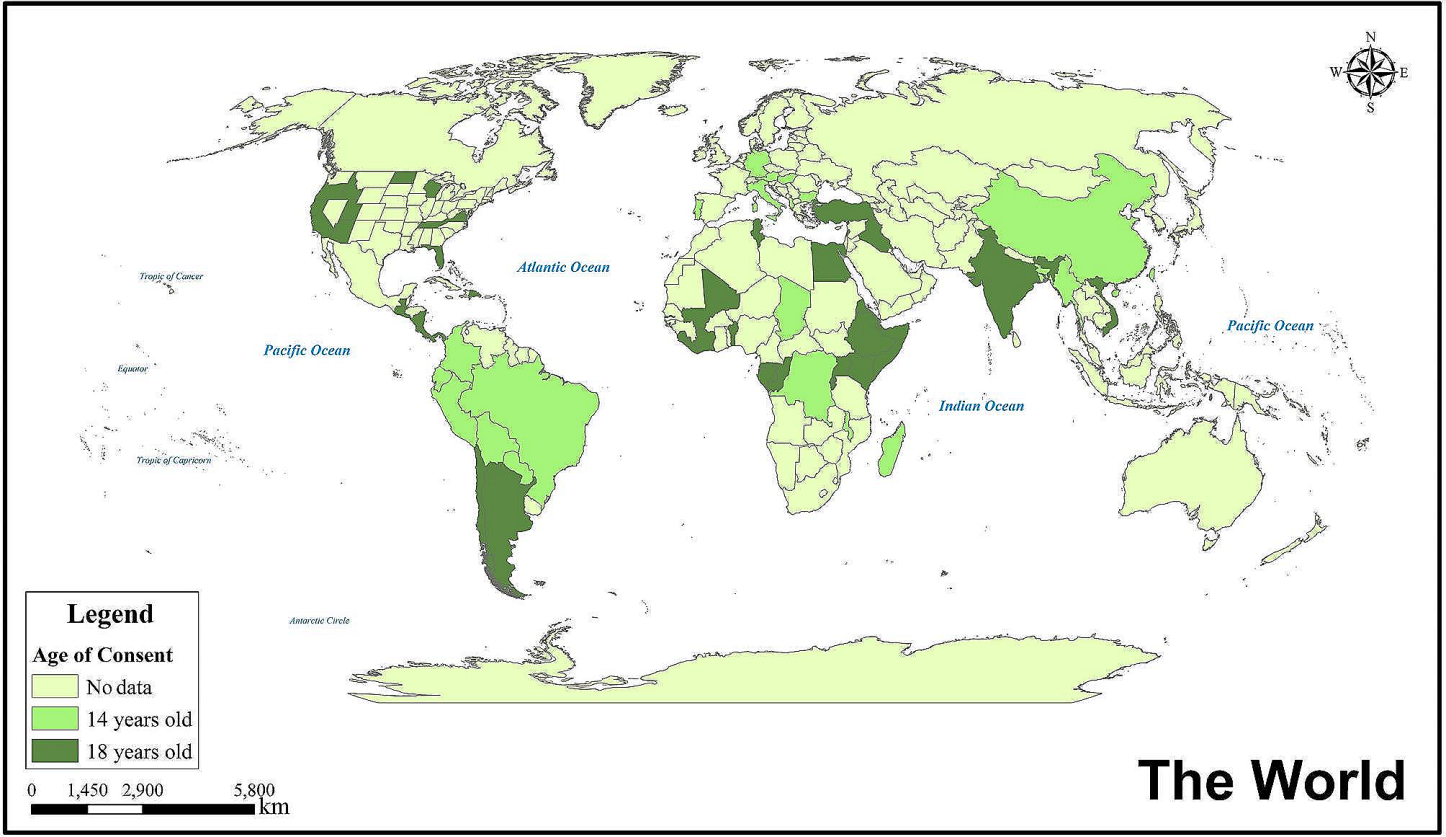
Countries or regions where the Age of consent is 14 or 18 years old

Age estimation is a vital component of personal identification and holds significant importance in forensic science, particularly when determining whether an adolescent meets the age of consent. “Age of consent” refers to the legally established age at which an individual is considered capable of providing consent for sexual activity. In cases involving potential sexual activity with a minor, personal identification becomes paramount in establishing whether an adolescent involved has reached the legal age of consent. Therefore, if a person above the legal age engages in sexual activity with an underage partner, such behavior may be considered statutory rape. Most countries/regions require individuals to be at least 14 years old to engage in sexual activity(https://worldpopulationreview.com/country-rankings/age-of-consent-by-country). Currently, 28 countries have set the “age of consent” at 14 years old, while 40 countries have set it at 18 years old. In the United States, the age of consent varies by state, with 11 states setting it at 18 years old (Fig. 1).

Teeth are unique among individuals, possessing individual characteristics that can be used for identification and individual profiling. Furthermore, dental development is closely related to age. Digital orthopantomographs (OPGs) are a common imaging technique that provides a panoramic view of the intraoral dental structures [1–9]. Traditional methods of age estimation rely on the development of seven teeth in the mandible (excluding third molars) [10]. However, difficulties arise when using these methods to estimate ages of 14 and 18 years old since most teeth have completed development by these ages, with only the apical foramina of the second and third molars remaining open [11].

Recent, research has shown a close correlation between the development of the periodontal ligament and age [12–20] Since Olze et al. introduced this concept in 2010 for individuals aged 18 and above [21], researchers have been exploring similar approaches. Guo et al. noted limitations in using third molars for age determination in the Chinese population due to delayed development [22–24]. This study proposes utilizing periodontal ligament visibility in second molars (PL_2M_) alongside other factors to address these limitations and improve accuracy, particularly in the Chinese population.

Traditional age estimation methods in dental medicine rely heavily on subjective observations, leading to biases and limitations. To mitigate these shortcomings, this study integrates machine learning (ML) techniques to enhance accuracy and objectivity. ML algorithms, trained on extensive datasets, can establish semi-automated age estimation models, leveraging global features and statistical patterns to improve accuracy [25–27].

Therefore, the main objectives of this study are to evaluate the third molar development using the Demirjian method (Demirjian_3M_), measure the third molar development index (I_3M_) using the Cameriere method [28], and assess PL_2M_. These measurements will be combined with various ML models, including random forest (RF), decision tree (DT), support vector machine (SVM), K-nearest neighbor (KNN), Bernoulli Naive Bayes (BNB), and logistic regression (LR), to predict whether adolescents have reached the age of 14 or 18. By integrating ML methods into age estimation, this research may have significant implications for personal identification, legal proceedings, and the establishment of effective age-related policies and regulations.

## Materials and methods

### Samples

All three aforementioned methods were conducted following appropriate operational guidelines and regulations. The research was approved by the Independent Ethics Committee of Shanghai Ninth Hospital, which is affiliated with Shanghai Jiao Tong University School of Medicine (Approval No. SH9H-2019-T75-3). The informed consent requirement for this study has been waived by the ethical committee, as the imaging data were collected from previously treated outpatients at our institution, and these data have been anonymized to ensure they cannot be linked to individual subjects.

The OPGs were captured using the KODAK 8000 C Panoramic and Cephalometric Digital Dental X-ray Machine. OPGs that did not include mandibular third molars were excluded from this study since the required data could not be obtained. Additionally, the exclusion criteria encompassed cases involving endodontic treatment for the mandibular third molar or second molar, poor-quality OPGs, and any related diseases that could affect jaw development.

According to the aforementioned conditions, this retrospective study analyzed OPGs obtained from a sample of 665 children aged 12 to 20 from eastern China. The sample consisted of 340 males and 325 females, who were divided into nine age groups according to their chronological age. The OPGs were categorized based on age and sex (Table 1).

**Table 1:** Age groups and sex distribution

Age Group	Female	Male	Total
12.00-12.99	35	37	72
13.00-13.99	43	38	81
14.00-14.99	41	40	81
15.00-15.99	37	40	77
16.00-16.99	37	38	75
17.00-17.99	29	33	62
18.00-18.99	34	47	81
19.00-19.99	36	42	78
20.00-20.99	33	25	58
Total	325	340	665

### Methods

To begin with, all OPGs were anonymized and numbered with Arabic numerals before the measurement and analysis.

First, the Demirjian method was used to assess the development stage of the mandibular third molar (Demirjian_3M_). According to the Demirjian classification, the mineralization degree of the mandibular third molar was evaluated in eight stages, labeled from A to H.

**Fig. 2:**
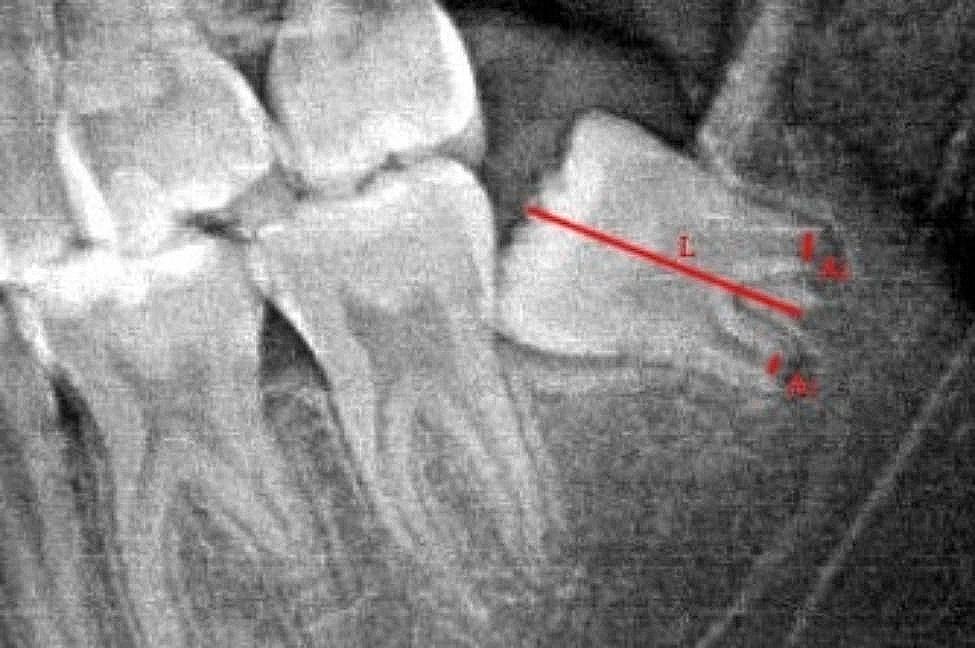
When measuring mandibular third molars with multiple roots, here is an example of a tooth with two roots. In this case, A represents the sum of the distances between the open root apices on the inner side (A = A_1_ + A_2_), and L represents the length of the third molar

Next, the Cameriere method was employed to measure the mandibular third molars (I_3M_). The measurement involved determining the distance of the root apex to the inner side (A) and the length of the tooth (L), and dividing the former by the latter to obtain I_3M_. In cases where the third molar had multiple roots, the distances of the root apices to the inner side were measured separately (A_1_ and A_2_), and their sum was recorded as A (A = A_1_ + A_2_). In other words, if the third molar was fully developed and the root apex was closed, I_3M_ was recorded as 0. Otherwise, I_3M_ was calculated as A divided by L (I_3M_ = A/L, Fig. 2).

**Fig. 3:**
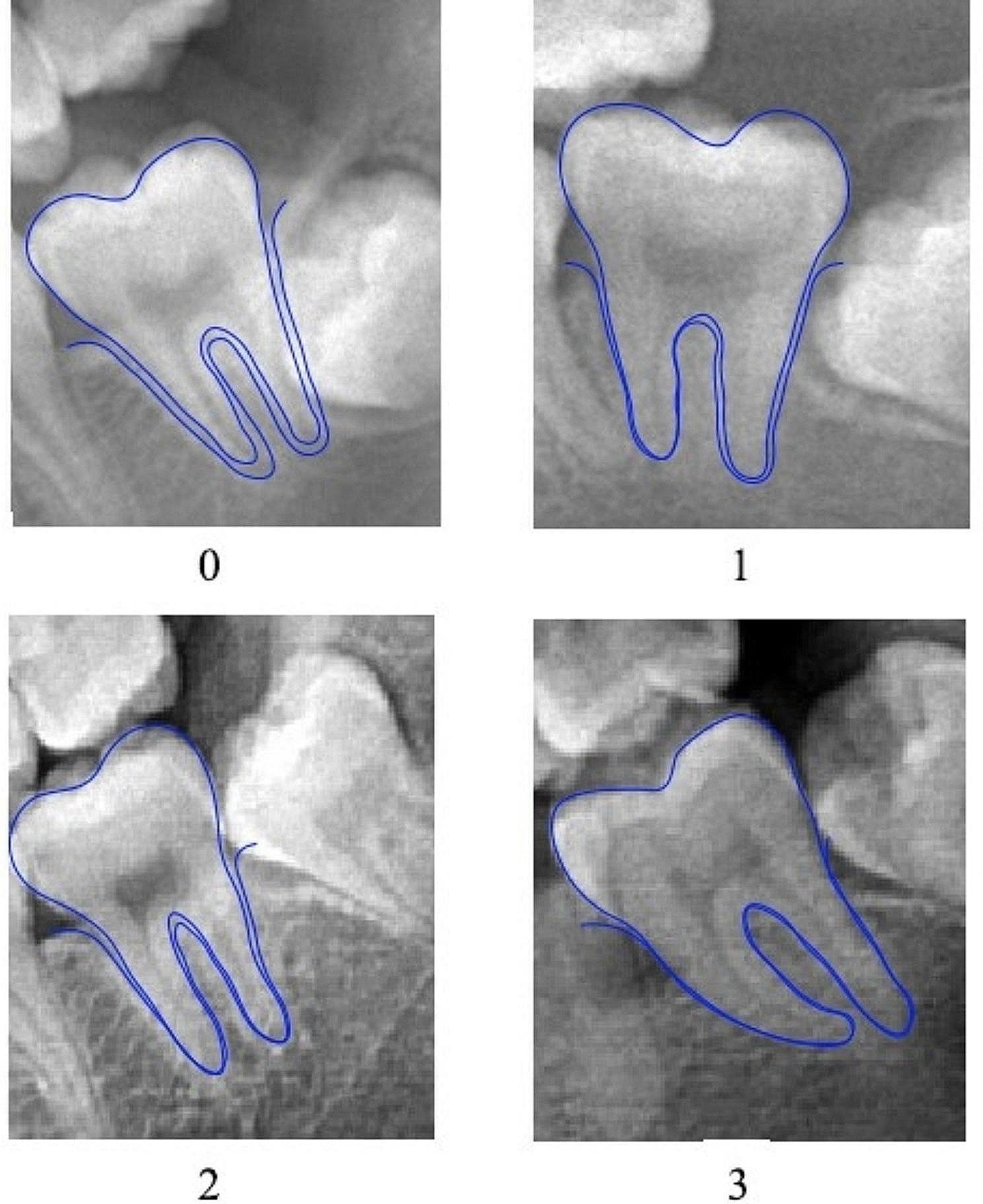
Pictures of the stages of radiographic visibility of the periodontal ligament in second molars

Finally, the visibility of the periodontal ligament between the tooth root and its socket of the mandibular second molar was evaluated (near the middle of the mesial root and the middle of the distal root). Based on Olze’s classification of periodontal ligament development, it could be categorized into four stages, as follows (Fig. 3). Table S1 provides a stage description for the periodontal ligament development.

After training, two observers independently evaluated OPGs without knowing any biological or identity information except for the subject codes. The observers utilized the three methods mentioned earlier to assess 20 randomly selected OPGs for inter-observer consistency.

As previously mentioned [29, 30], reliability assessment utilized Cohen’s Kappa for Demirjian_3M_ and PL_2M_ due to their ordinal nature. Conversely, the Intra-class correlation coefficient (ICC) was employed for the I_3M_ method.

To address the binary classification problem of determining whether a subject was over 14 or 18 years old based on sex (coded as male = 1, female = 0), PL_2M_ (coded as 0, 1, 2, 3), I_3M_, and Demirjian_3M_ (coded as A = 1, B = 2, …, H = 8) variables, six ML models were trained. The chronological age served as the classification criterion. The performance of the models was evaluated using the well-known K-fold cross-validation method, with k set to 5 in this study. To prevent overfitting, a 20% validation dataset was utilized during hyperparameter optimization.

The hyperparameters of the models were fine-tuned by exploring multiple combinations using the GridSearchCV function. Supplementary Table S2 provides a description of the hyperparameters that were tuned to obtain the best model.

### Statistical analysis

In this study, the accuracy of the aforementioned methods was compared using sensitivity, specificity, overall accuracy, and post-test probabilities. The chronological age of the samples was obtained by subtracting the birth date from the OPGs’ acquisition date. The inferred age was considered as the binary response variable, while the predictor variables included sex, PL_2M_, I_3M_, and Demirjian_3M_.

A generalized linear model, specifically a logistic model, was utilized to predict whether an individual was under 14 years old (E = 0) or over 14 years old (E = 1).

The testing was conducted to obtain the sensitivity p1 (the proportion of verified T = 1 events among adolescents aged 14 years or older) and the specificity p2 (the proportion of verified T = 0 events among adolescents under 14 years old). The calculation formula for post-test probabilities was derived from Bayes’ theorem:

$$\text{p}=\frac{{S}_{e}{p}_{0}}{{S}_{e}{\times p}_{0}+\left(1-{S}_{p}\right)\left(1-{p}_{0}\right)} \left(1\right)$$

Where p represents the post-test probability, and p0 denotes the proportion of individuals aged 12 to 20 who have actual ages of 14 or greater. This proportion was calculated to be 0.824 overall. The assessment for individuals aged 18 follows the same procedure as described above. Specifically, for individuals aged 18, the value of p is 0.403. The estimation of p0 was based on demographic data obtained from the National Bureau of Statistics of the People’s Republic of China (http://www.stats.gov.cn/tjsj/pcsj/).

Data analysis and the creation of related figures were performed using SPSS 25.0 (IBM Corp. Released 2017. IBM SPSS Statistics for Windows, Version 25.0. Armonk, NY: IBM Corp.), PyCharm 2021, and Python 3.8.2. The statistical significance level was set at 5%.

## Results

For I_3M_, the intraclass correlation coefficient (ICC) for the intrarater agreement was 0.926. Meanwhile, the ICC for the interrater agreement was 0.865. For PL_2M_ and Demirjian_3M_, the intrarater Kappa was 0.895, and the interrater Kappa was 0.828.

**Fig. 4:**
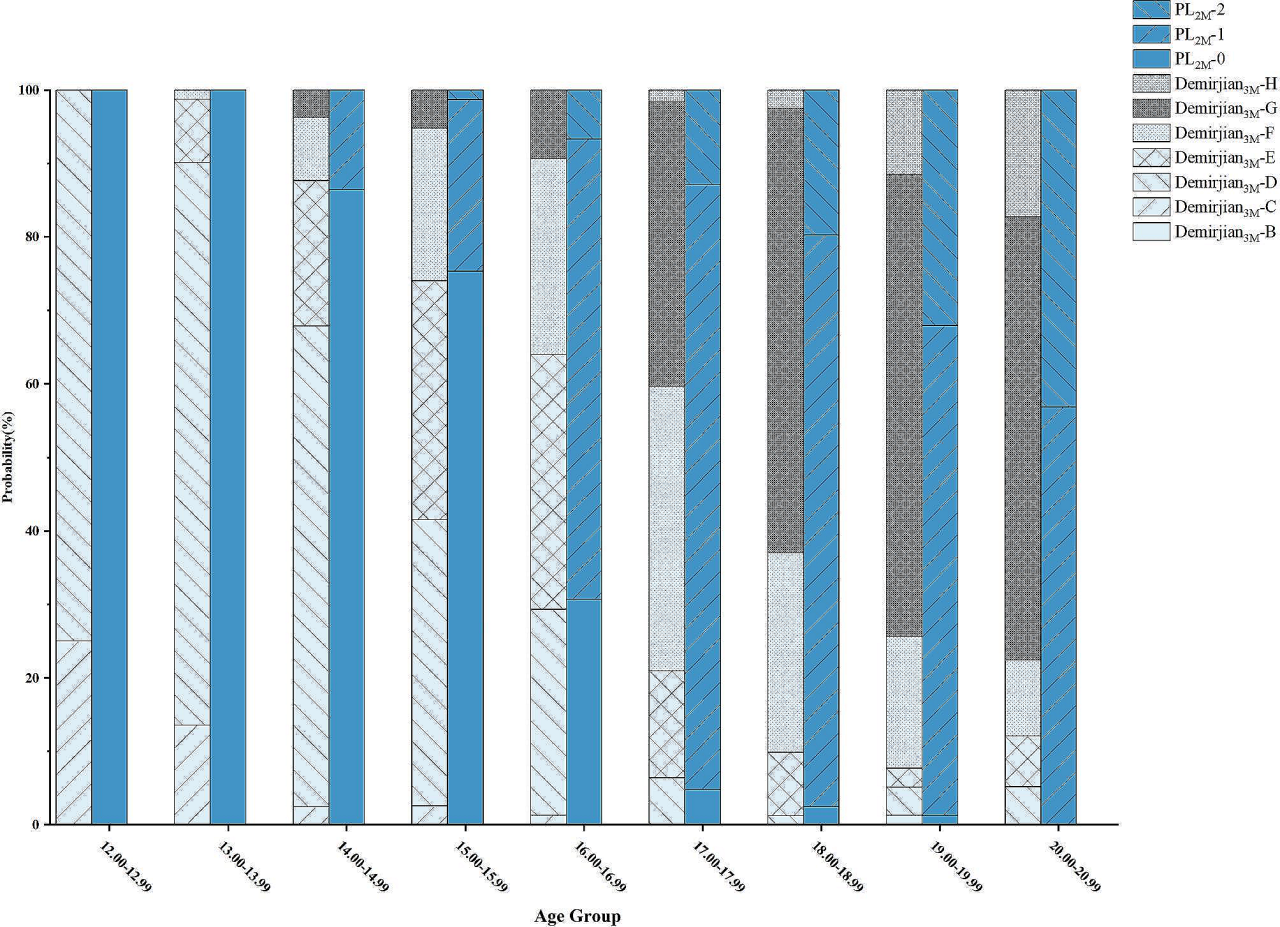
Percentage stacked bar chart of Demirjian_3M_ and PL_2M_ across different age groups

In Fig. 4, it is evident that the distribution of Demirjian_3M_ stages varies across different age groups, with stages D and G being the most prevalent and stages C and B less common. The proportions of stages E, F, and H fluctuate across age groups. Notably, there is an increasing trend in the proportion of stages E and F with age, indicating a progressive maturation of dental development in children over time.

Moreover, the stacked bar chart (Fig. 4) also shows a shift in the distribution of PL_2M_ stages with age. Stage 0 predominates in younger age groups, while stages 1 and 2 become more prevalent in older age groups, suggesting a progressive maturation of dental development as individuals grow older.

**Fig. 5:**
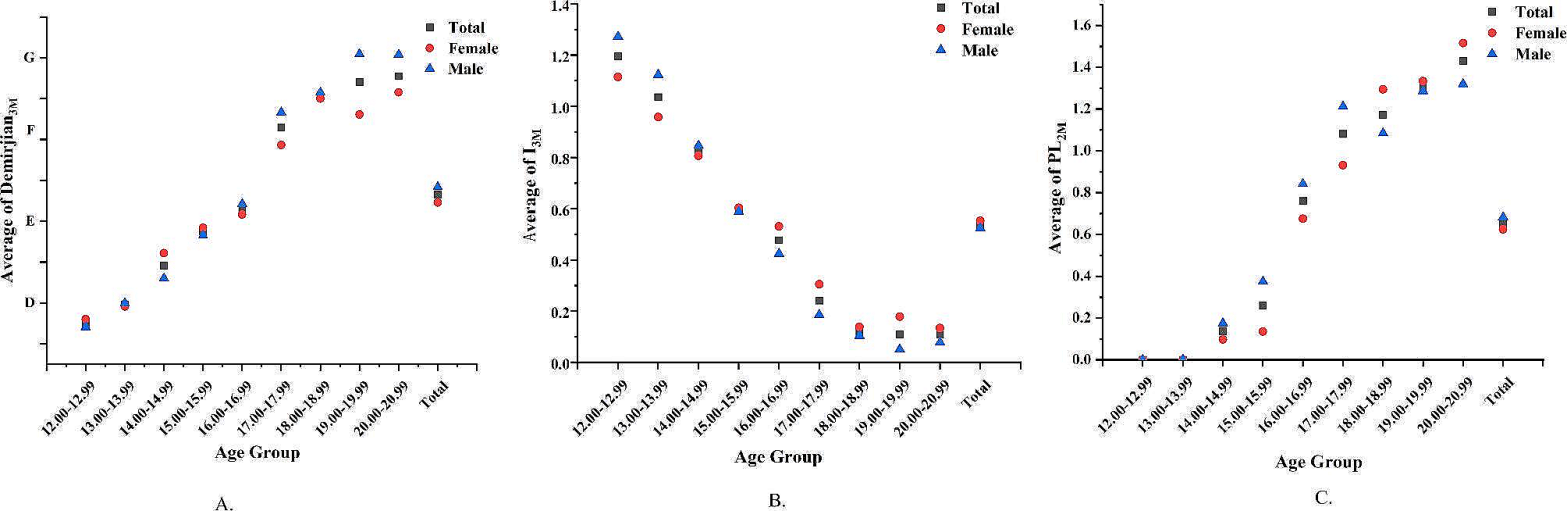
Scatterplot of average Demirjian_3M_, I_3M_, and PL_2M_ across different age groups

Moving on to the scatterplot chart (Fig. 5), there is a general downward trend in I_3M_ scores as age increases, indicating the gradual maturation of tooth development. This decline in I_3M_ scores can be observed for both females and males. In contrast, Demirjian_3M_ scores exhibit an upward trend with age, indicating increasing tooth development across different age groups. These trends in I_3M_ and Demirjian_3M_ scores support the concept of progressive dental development and maturation as individuals age.

**Fig. 6:**
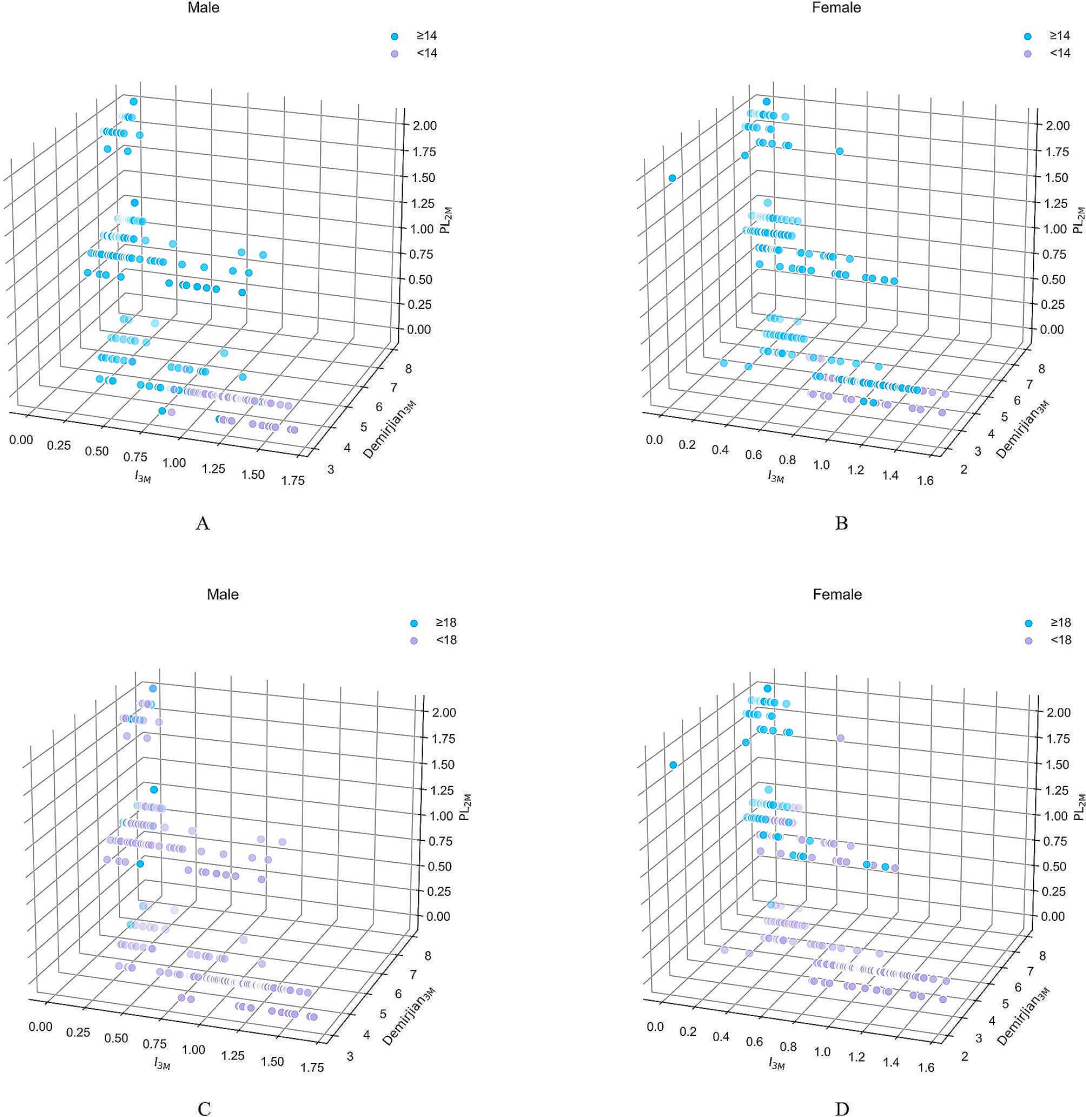
3D scatter plots depict the relationship between age and dental maturity indicators (I3M, Demirjian3M, and PL2M) for (A) males and (B) females at the age of 14, and for (C) males and (D) females at the age of 18

The 3D scatter plots (Fig. 6A) and (Fig. 6B) provide a visual representation of the relationship between age, I_3M_, Demirjian_3M_, and PL_2M_ for males and females, respectively. Each data point represents an individual, and their positions in the plot indicate the values of the variables. The scatter plots (Fig. 6C) and (Fig. 6D) focus specifically on 18-year-old males and females, showcasing the relationships between these variables for these specific groups.

As actual age increases, there is a general trend of increasing values for PL_2M_ and Demirjian_3M_, while I_3M_ values tend to decrease, reflecting tooth maturation. The separation between the blue and purple dots in the scatter plots signifies that these markers can be used to differentiate samples of individuals aged 14 or 18 and above or below.

The Table 2 provides a comparison of the performance of different ML models (LR, RF, DT, KNN, BNB) in determining whether an individual is above the age of 14. The sensitivity, specificity, accuracy, and post-test probability metrics are evaluated for each model. Comparing these models, we observe that LR and BNB have the highest sensitivity (0.91 and 0.909) and accuracy (0.83) among all the models. They also yield similar post-test probabilities of 0.935. RF and DT models have slightly lower sensitivity (0.691 for RF and 0.687 for DT) and post-test probabilities but still perform reasonably well (0.931 for both). KNN model shows the lowest accuracy among the models evaluated (0.814).

**Table 2:** Sensitivity, specificity, post-test probability, and accuracy of six machine learning models for assessing the age of 14

	LR	RF	DT	SVM	KNN	BNB
Sensitivity	0.910	0.893	0.897	0.905	0.910	0.909
Specificity	0.705	0.691	0.687	0.693	0.700	0.703
Post-test probability	0.935	0.931	0.931	0.932	0.934	0.935
Accuracy	0.830	0.826	0.818	0.819	0.814	0.830

LR, Linear regression; RF, random forest; DT, decision tree; SVM, support vector machine; KNN, K-nearest neighbor; BNB, Bernoulli Naive Bayes

Table 3 presents the comparison of different models for determining whether an in-dividual is above the age of 18. When assessing sensitivity, which measures the ability to correctly identify individuals over 18, LR achieves a sensitivity of 0.776, followed closely by BNB with a sensitivity of 0.778. However, RF shows the lowest sensitivity at 0.770. For specificity, which measures the ability to correctly identify individuals under 18, all models demonstrate relatively high values ranging from 0.904 to 0.909. LR, SVM, KNN, and BNB models show similar performance in terms of specificity. In terms of accuracy, all models achieve values ranging from 0.796 to 0.807, with LR having the highest accuracy of 0.806. The post-test probabilities, reflecting the likelihood of an individual being above 18 given a positive test result, range from 0.842 to 0.852, indicating a moderate level of confidence in the predictions.

**Table 3:** Sensitivity, specificity, post-test probability, and accuracy of six machine learning models for assessing the age of 18

	LR	RF	DT	SVM	KNN	BNB
Sensitivity	0.776	0.770	0.761	0.760	0.769	0.778
Specificity	0.909	0.905	0.904	0.909	0.909	0.908
Post-test probability	0.851	0.844	0.842	0.851	0.852	0.851
Accuracy	0.806	0.796	0.796	0.807	0.804	0.806

LR, Linear regression; RF, random forest; DT, decision tree; SVM, support vector machine; KNN, K-nearest neighbor; BNB, Bernoulli Naive Bayes

Comparing Tables 2 and 3, differences in the performance of models for determining whether an individual is above the age of 14 versus above the age of 18 are evident. While LR and BNB models demonstrate relatively high sensitivity and accuracy in both age groups (for age 14, LR sensitivity is 0.91, BNB is 0.909, LR and BNB accuracy are both 0.830; for age 18, LR sensitivity is 0.776, BNB is 0.778, LR and BNB accuracy are both 0.806), other models show varying levels of performance. Notably, RF exhibits lower sensitivity in the age 18 classification task (0.770) compared to age 14 (0.893). Additionally, specificity values remain consistently high across all models in both age groups.

## Discussion

Age estimation is pivotal in personal identification, especially in determining if an individual has reached the legal age of consent, typically defined as 14 or 18 years old. This determination carries significant forensic implications as it directly impacts various legal and social considerations. Furthermore, accurate age assessment holds immense importance in legal proceedings, underlining the critical role of age estimation across different contexts.

This study aims to pioneer the integration of ML with the evaluation of PL_2M_, Demirjian_3M_, and I_3M_, to determine whether an individual has reached the age of 14 or 18. This comprehensive approach improves the accuracy and reliability of age estimation, considering various developmental aspects simultaneously. By incorporating ML, this study aims to improve the accuracy and objectivity of age estimation. Human subjectivity and potential biases can be minimized, leading to more consistent and reliable results.

The LR and BNB models exhibit higher sensitivity and accuracy in predicting ages of 14 and 18. LR excels in capturing linear relationships between features and labels, particularly advantageous in this study given the likely linear associations between features like gender and dental assessment indicators (e.g., Demirjian_3M_, PL_2M_, and I_3M_) with chronological age [31]. LR efficiently incorporates these relationships, resulting in heightened predictive accuracy [32]. Conversely, BNB’s assumption of feature independence, rooted in Bayes’ theorem, proves advantageous despite potential correlations among dental assessment indicators [33]. This assumption enables BNB to effectively manage these features, swiftly converging and learning the data distribution, thereby achieving notable predictive sensitivity and accuracy [34].

This study addresses the limitations of traditional human-based methods, such as SPSS, in predicting whether adolescents reach the age of 18. Previous approaches often relied on a single variable alongside gender, resulting in potential biases and incomplete results. Furthermore, the inconsistent performance of the I_3M_ = 0.08 threshold used across regions to determine whether individuals have reached the age of 18 highlights the challenges of manual assessment. For instance, in Turkey, the accuracy rates for I_3M_ = 0.08 are 0.847 for males and 0.846 for females [35]; in the northern region of Brazil, the accuracy rates are 0.731 for females and 0.800 for males, with an overall rate of 0.761 [36]; whereas for the African Black population in Botswana, the accuracy rates are 0.91 for males and 0.92 for females [37]. In contrast, ML techniques offer a solution by incorporating multiple predictor variables and adapting to regional variations in data [31]. ML algorithms excel in handling complex interactions between variables, detecting patterns, and accommodating non-linear relationships, thus constructing more comprehensive models [31, 32]. Additionally, ML models are robust to violations of traditional statistical assumptions, ensuring accurate analyses even with intricate datasets [38].

In future research, it is possible that additional growth and developmental factors will be incorporated to enhance the accuracy of determining the age of consent. By expanding the scope of investigation to include a broader range of biological, physiological, and environmental factors that influence development, researchers can strive to refine and improve the predictive models used in assessing age of consent. By considering a more comprehensive set of variables, it may be possible to achieve greater precision and reliability in determining the age at which individuals can provide informed consent.

## Conclusion

Overall, the results indicate that the ML models are effective in age estimation, especially for individuals above the age of 14. However, the performance decreases slightly when estimating ages above 18, suggesting the need for additional factors or models to enhance accuracy in age determination for older individuals. Therefore, the integration of dental assessments and machine learning methods offers an accurate and reliable approach for age prediction and personal identification.

### Electronic supplementary material

Below is the link to the electronic supplementary material.

Supplementary Material 1

## Data Availability

The datasets used and/or analyzed during the current study available from the corresponding author on reasonable request.
