# Supplementary material for: Predicting chronological age of 14 or 18 in adolescents: integrating dental assessments with machine learning
Source: BMC Pediatr. 2024 Apr 10;24:248. doi: 10.1186/s12887-024-04722-1 (PMC11005269; doi:10.1186/s12887-024-04722-1)
Supplement: Supplementary file 1 — Supplementary Material 1 [file 12887_2024_4722_MOESM1_ESM.docx]

Supplementary Table S1 A stage description for the periodontal ligament development based on Olze’s classification

| Stage 0 | *The entire periodontal ligament around the lower left third molar is visible, representing 100% visibility* |
| --- | --- |
| Stage 1 | *The visibility of the periodontal ligament (PLV) is between 75% and 50% of its total area. This is determined by mentally summing up the pattern of PLV across both the mesial and distal roots.* |
| Stage 2 | *PLV ranges from 50% to 25% of its total area around the lower left third molar. This summation is done considering both the mesial and distal roots.* |
| Stage 3 | *There is no discernible PLV or it is extremely minimal. In other words, 100% of the periodontal ligament of the lower left third molar has disappeared.* |

Supplementary Table S2 List of the tuned hyperparameters for each Machine Learning algorithm. For each hyperparameter, the values inside square brackets were explored by Grid Search.

| K-Nearest Neighbors (KNN) | *n_neighbors*: the number of neighbors or K to use [12 to 20] *weights*: the weight function used in prediction [uniform or distance]  *algorithm*: type of algorithm used to compute the nearest neighbors [ball tree, kd tree or brute] |
| --- | --- |
| Decision Tree (DT) | *criterion*: the function to measure the quality of a split in the tree [mse, Friedman mse or mae]  *max_depth*: the maximum depth of the tree [6 to 8]  *max_features*: the number of features to consider when looking for the best split at a node [auto, sqrt, log2]  *min_samples_split*: the minimum number of samples required to split an internal node [2 to 5]  *min_samples_leaf:* the minimum number of samples required to be at a leaf node [2 to 5]  *splitter*: the strategy used to choose the split at each node [best or random] |
| Bernoulli Naive Bayes (BNB) | *alpha_1*: shape parameter for the Gamma distribution prior over the alpha parameter [1e^-15^, 1e^-10^, 1e^-8^, 1e^-4^, 1e^-3^, 1e^-2^, 1, 5, 10, 20] *alpha_2*: inverse scale parameter (rate parameter) for the Gamma distribution prior over the alpha parameter [1e^-15^, 1e^-10^, 1e^-8^, 1e^-4^, 1e^-3^, 1e^-2^, 1, 5, 10, 20] *lambda_1*: shape parameter for the Gamma distribution prior over the lambda parameter [1e^-15^, 1e^-10^, 1e^-8^, 1e^-4^, 1e^-3^, 1e^-2^, 1, 5, 10, 20] *lambda_2*: inverse scale parameter (rate parameter) for the Gamma distribution prior over the lambda parameter [1e^-15^, 1e^-10^, 1e^-8^, 1e^-4^, 1e^-3^, 1e^-2^, 1, 5, 10, 20] |
| random forests (RF) | n_estimators: the number of trees in the forest [5, 10, 15, 20, 30, 40, 50],  max_depth: the maximum depth of a tree [6 to 8]  max_features: the number of features to consider when looking for the best split at a node [auto, sqrt, log2]  min_samples_split: the minimum number of samples required to split an internal node [2 to 5]  min_samples_leaf: the minimum number of samples required to be at a leaf node [2 to 5]  bootstrap: whether bootstrap samples are used when building trees [True, False] |
| Support Vector Machine (SVM) | kernel: the kernel type used in the algorithm [polynomial, linear, rbf]  gamma: kernel coefficient [0.0001, 0.0005, 0.001, 0.01, 0.05, 0.1]  C: the regularization parameter. The higher the parameter the lower the regularization strength [1 to 10] |
| Logisitic Regression(LR) | penalty: the choice of regularization term [l1,l2]  solver: the solver type used in the algorithm [liblinear, newton-cg, lbfgs] |
